# A Qualitative Study of Pregnant Women’s Perspectives on Antibiotic Use for Mom and Child: Implications for Developing Tailored Health Education Interventions

**DOI:** 10.3390/antibiotics9100704

**Published:** 2020-10-15

**Authors:** Lynn Y. Chen, Elizabeth Flood-Grady, Austen Hentschel, Lauren Wright, Rahma Mkuu, Alyson Young, Magda Francois, Josef Neu, Leslie A. Parker, Elizabeth Shenkman, Janice L. Krieger, Dominick J. Lemas

**Affiliations:** 1 Department of Health Outcomes and Biomedical Informatics, College of Medicine, University of Florida, Gainesville, FL 32611, USA; lychen20@ufl.edu (L.Y.C.); austenlhentschel@ufl.edu (A.H.); laurenwright42@ufl.edu (L.W.); rmkuu@ufl.edu (R.M.); alys.yng@ufl.edu (A.Y.); magdafrancois@ufl.edu (M.F.); eshenkman@ufl.edu (E.S.); 2 STEM Translational Communication Center, College of Journalism and Communications, University of Florida, Gainesville, FL 32611, USA; efloodgrady@ufl.edu (E.F.-G.); janicekrieger@ufl.edu (J.L.K.); 3 Clinical Translational Science Institute, University of Florida, Gainesville, FL 32611, USA; 4 Department of Pediatrics, University of Florida Health, Gainesville, FL 32611, USA; neuj@peds.ufl.edu; 5 Department of Behavioral Nursing Science, University of Florida, Gainesville, FL 32611, USA; parkela@ufl.edu

**Keywords:** antibiotics, health decision-making, pregnancy, obstetrics, pediatrics

## Abstract

The overutilization of antibiotics during pregnancy and early life are associated with adverse health outcomes for mothers and infants. In this study, we explored pregnant women’s opinions and concerns of antibiotics and how perceptions may affect their health-related decision-making. We conducted 18 in-depth, semi-structured interviews with pregnant women and used the Health Belief Model (HBM) as a framework to analyze the data. We found that mothers generally understood the benefits of antibiotics and were aware that antibiotics are clinically effective for treating bacterial infections. Importantly, perceived barriers related to antibiotic use included concerns regarding the impact of antibiotics on breastfeeding efficacy, microbial health, and societal factors such as antimicrobial resistance. The prescription of antibiotics by a healthcare provider was a cue to action for women, as they trusted providers to recommend medications that were safe for them and their infants. Overall, mothers shared that receiving education on the effects of antibiotics would improve their self-efficacy and decision-making surrounding the use of antibiotics for treating illness. Implications for tailored perinatal health education interventions to enhance antibiotic use, knowledge, and decision-making are discussed.

## 1. Introduction

Antibiotics are among the most frequently prescribed medications for women during pregnancy [1] and children during early life [2]. Around 20–35% of women are given antibiotics during pregnancy [3,4,5] for reasons such as treatment for urinary tract infections (UTIs) and respiratory tract infections (RTIs) [6]. Although antibiotics can successfully treat infections, antibiotics can adversely affect one’s health. Many antibiotics are listed as possible teratogens for pregnancy [7], and the U.S. Food and Drug Administration requires prescription drug manufactures to label products and provide information about the risks and benefits of medications during pregnancy, lactation, and among men and women of reproductive age [8]. Evidence suggests antibiotics may affect the fetus’ microbiome in utero [9], and research on mother’s milk content also indicates that women can pass antibiotics taken during pregnancy to their child through their milk [10]. Antibiotic introduction among children during early age may be associated with life-long health deterrents, such as obesity [11] and asthma [12,13]. Given the duality of antibiotics’ effects in the body, women may face a difficult decision when considering usage for themselves during pregnancy and lactation alongside the potential effects antibiotics may have for their child.

Shared decision-making is a commonly used model in primary care that encourages a two-way exchange of information between healthcare providers and patients when discussing treatment [14]. Pregnant women have extra considerations when making health-related decisions, as they must take into account their health and the health of their unborn baby [15]. This dual decision-making process extends after delivery when women take responsibility for the health of their infants. To better understand women’s health decision-making during pregnancy and after delivery, evaluating their pre-existing beliefs and thought processes surrounding health and treatment is imperative. The Health Belief Model (HBM) is a psychological model of human behavior that provides a framework to understand how key health beliefs influence health behaviors [16]. The HBM takes into consideration people’s perceived susceptibility and perceived severity of illness (dimensions of threat), perceived barriers and perceived benefits of a behavior (dimensions of “net benefits”), cues to action (stimulus to decide to act), and self-efficacy (the confidence or ability to perform a behavior) as factors influencing behaviors [17]. Specifically, we used the HBM to explore how specific multi-dimensional constructs inform pregnant women’s decisions related to antibiotics use. The utility of the HBM is in its ability to provide an explanatory framework to understand how expectations for treatment and threats of illness are related to health decision-making. Prior studies have used the HBM to study the decision-making of pregnant women on dietary patterns [18], undergoing amniocentesis [19], and mode of delivery [20]. The HBM has also been used to explore antibiotic decision-making for individuals [21,22]. However, information regarding maternal opinions of antibiotics during and after pregnancy and their decision-making processes for their children remains limited.

The purpose of this paper is to utilize the HBM as a theoretical framework to understand pregnant women’s perceptions of antibiotics and decision-making processes surrounding antibiotic use for themselves and their infants. According to the HBM, individuals who believe they are susceptible to disease (perceived susceptibility), that the disease may have undesirable effects (perceived severity), and that the positives (perceived benefits) outweigh the risks (perceived barriers) in a particular health situation are more likely to take action to receive medical care [16]. One’s decision-making also relies on their perceived self-efficacy, which is an individual’s belief in their ability to successfully take action to receive the desired treatment [23]. The HBM also accounts for cues to actions, which are defined as separate events that are not associated with one’s perceptions that may trigger treatment engagement [16]. We use the HBM and qualitative methods to characterize pregnant women’s perceptions of antibiotics and decision-making processes surrounding antibiotic use for themselves and their infants and to identify and describe their preferences for antibiotics counseling.

## 2. Results

### 2.1. Participant Demographics

Table 1 describes the variation in demographics of participants that completed the interview (*n* = 18). The women in our study were between the ages of 22 and 39 years, the majority of which were between 31 and 40 years of age (*n* = 10, 56%). Among participants, more than half (*n* = 11, 61%) were pregnant with their first child, nine (50%) held a professional or graduate degree, and five (28%) reported taking an antibiotic while pregnant.

### 2.2. Factors Involved in Perceptions of Antibiotics and Decision-Making: The Health Belief Model

**Figure 1:**
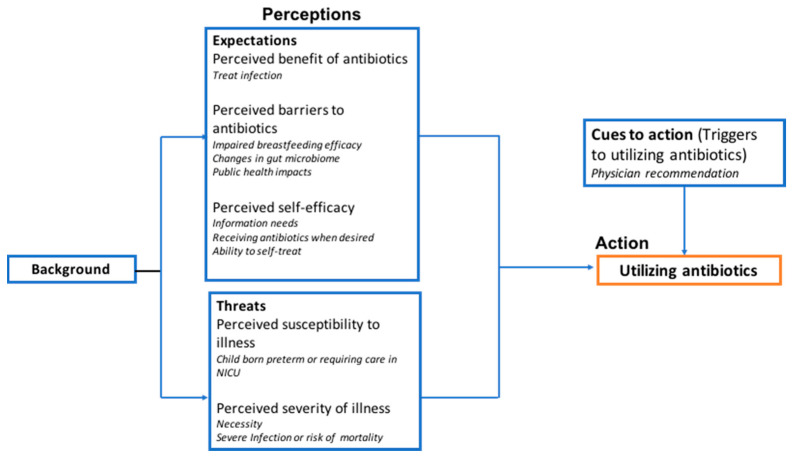
The Health Belief Model: Thematic summary of factors that impact antibiotic decision-making.

The components of the HBM used to analyze how pregnant women make decisions related to antibiotic utilization included perceptions of the expectations of antibiotic use and threats of illness, as well as cues to action. The expected relationship of these factors is shown in Figure 1. Components involved in expectations of antibiotic use include perceived benefits (Section 2.2.1) and barriers (Section 2.2.2) of antibiotics and perceived self-efficacy (Section 2.2.3) surrounding antibiotic use. Threats of illness are comprised of perceived susceptibility (Section 2.2.4) and severity of illness (Section 2.2.5). The final portion of the HBM includes cues to action (Section 2.2.6), which are events that trigger antibiotic use. Below, we have expanded on these sub-themes and provided participant quotes. We completed subgroup analysis where appropriate.

#### 2.2.1. Expectations of Antibiotic Use: Perceived Benefits of Taking Antibiotics

Overall, we found that women showed an understanding that antibiotics are effective in treating illnesses and fighting infections. Statements that indicated perceptions of antibiotic benefit included the following:

*“When I think of antibiotics, I think of like a way to get better from being sick.”*

—PRG005

*“If you’re sick and you’re getting treated, yeah, it’s awesome, if you’ve got a reason to take them, which you know she’s like, “Okay, you’re not getting any better, it’s not viral, try the antibiotics and see if it’ll help.”*

—PRG018

Despite a general understanding about the benefits of antibiotic use, the majority of women indicated negative sentiments surrounding antibiotics. Thirteen participants stated that they did not favor antibiotics and would avoid taking them if possible:

*“In general, I’m not a big fan of antibiotics and I try not to take them.”*

—PRG002

#### 2.2.2. Expectations of Antibiotic Use: Perceived Barriers of Taking Antibiotics

Barriers were characterized by women’s beliefs about the risks associated with using antibiotics and how antibiotics would affect their children. Women articulated both tangible and psychological barriers to antibiotic use. Participant’s concerns about antibiotic use centered on their potential to impair breastfeeding efficacy, lead to changes in gut microbiome, and impact public health.

Eight participants were concerned with the potential for antibiotics to affect breastfeeding efficacy. Breastfeeding efficacy refers to a mother’s ability to successfully breastfeed her child for an extended period of time. When asked, half of the women believed that the use of antibiotics by herself or her child might prevent them from reaching their breastfeeding goals. Participants expressed concerns about antibiotics causing thrush in the mouth for their child or affecting the nutrients that they can provide for their child. Respondents noted:

*“It could in the same way like with thrush in the mouth… I think in that sense, it could impact the breastfeeding goals.”*

—PRG016

*“The way that antibiotics works is it kills bad—the bad bacteria, but it also kills the good bacteria as well. I think, it can impact the ability of—for the breastmilk to actually give the nutrients that it needs to the child.”*

—PRG013

Participants (*n* = 8) also reported that potential changes to the infant gut microbiome was a barrier to antibiotic usage. Respondents understood that antibiotics could alter the gut microbiome and have lasting impacts on overall health. Women also expressed concerns that antibiotic usage at an early age could disrupt the development of the infant gut microbiome, which could affect their child’s immune system and digestive health at older ages. Statements coded regarding changes in gut microbiome included:

*“Antibiotics mess up your gut health, and that gives you like other issues and other ailments and other things. It’s just like a domino effect.”*

—PRG020

*“I definitely want to know how [antibiotics] affect a baby’s health or digestion cuz I’m sure it’s not just like in the moment. It’s gonna show up later on.”*

—PRG007

Concerns of antibiotic overutilization and resistance were mentioned by participants (*n* = 5) as reasons to avoid antibiotics usage. Participants were concerned that antibiotics are generally overprescribed by healthcare providers and often used prematurely to expedite treatment and may not be necessary in all cases. Women concerned about public health recounted previous negative experiences with healthcare providers who, they believed, inappropriately prescribed antibiotics. One respondent noted:

*“I just feel like they just prescribe something, because they learned how to do that. So, I think they’re overutilized. I think if you’re sick, instead of getting to the root of the problem, they’ll just be like, ‘Oh, take this antibiotic and cure that…’ and not cure the actual root of the issue.”*

—PRG020

Participants were also concerned with the overutilization of antibiotics and that it may lead to antibiotic resistance and the development of superbugs. One woman stated,

*“Well, and there’s all the resistance issues and everything which is why … I’m like, I’m gonna do my part to help with that by not taking it unless I absolutely need it.”*

—PRG018

#### 2.2.3. Expectations of Antibiotic Use: Perceived Self-Efficacy Surrounding Antibiotic Use

Perceived self-efficacy refers to the conviction that one can take action and/or make a decision about taking antibiotics. Factors that contributed to women’s feelings of self-efficacy to exercise control over taking antibiotics were information needs, receiving antibiotics when desired, and self-treatment using alternative medicine.

The majority of participants (*n* = 14) discussed their desire for information about antibiotics prior to use as a major factor in considering their decision to use antibiotics for themselves or their infant. Women’s needs included learning information about the pros and cons of antibiotic use and were interested in online resources and information from healthcare providers. Obtaining adequate information about the benefits and risks of antibiotics gave mothers confidence that they would be able to decide what was best for them and their child. Women said the following about their information needs regarding antibiotics:

*“As long as I know what’s going on in my child’s body or my body, then I don’t think that I’d have an issue with anything. I want to know how it’s affecting me and know exactly what that antibiotic is going to do for me and my child. So as long as I know that information and I have both the negatives and positives, I don’t see why not.”*

—PRG005

*“[I would like] some honest information about the benefits and risks, pros and cons, both, to be able to make it an informative decision.”*

—PRG011

Four participants described the ability to ask for and receive antibiotics when they desired as another way to take control of their health and the health of their children. Participants believed that they were taking an active role in relieving their child of pain and illness by asking their health care provider for antibiotics. These women stated:

*“Because I think a lot of times moms are just like, we need something to get the baby to feel better.”*

—PRG004

*“When your child is crying and in pain, you just want to do something.”*

—PRG002

Some women (*n* = 8) showed preferences toward using alternative methods, such as herbal and natural remedies, to mitigate illness. Respondents were confident in their ability to successfully treat illnesses using alternative methods, thus eliminating the need for antibiotics. Women expressed:

*“I’ll take antibiotics but if I can take like a ton of vitamin C and like get a lot of sleep, I would rather do that than affecting all the bio stuff in me.”*

—PRG007

*“If it is preventive or unnecessary, then let me try to do—something different because there are other ways that you can heal the body other than antibiotics.”*

—PRG013

Mothers also shared that they would not give their infant antibiotics while breastfeeding, as they believed that the nutrients passed through breastmilk would be effective alone in preventing and treating their infant of illnesses. One mother expressed this sentiment by saying:

*“I don’t think I would give my baby antibiotics if she’s sick or not because breastfeeding; you get all the nutrients you need, so you won’t get sick, so I probably won’t even like, “No. You can keep your medicine. I have got this.”*

—PRG006

#### 2.2.4. Threats of Illness: Perceived Susceptibility of Illness

Women’s beliefs about the perceived severity of the illness/infection and the need for antibiotics centered on having a child born preterm or infant in the Neonatal Intensive Care Unit (NICU).

Participants (*n* = 6) believed that children born preterm and those who required care in the NICU were more susceptible to developing an illness due to the underdevelopment of vital organs or increased exposure to pathogens in the unit. Women with infants in these situations said they would be more inclined to give their infants antibiotics:

*“I think a lot of my perspectives and my crazy crunchy remedies would change if I had a preemie. I’d be like, “Get me to the nearest hospital. Give me all the drugs.” So, I think it’s all a lot of your situation.”*

—PRG020

#### 2.2.5. Threats of Illness: Perceived Severity of Illness

Women’s beliefs about the perceived severity of the illness/infection and the need for antibiotics were based on necessity and the severity of the infection or risk of mortality. Women perceived illness threats in similar ways.

Antibiotics as a “necessity” was widely stated by women (*n* = 12) as a condition in which they would take antibiotics or give antibiotics to their children. Mothers loosely defined necessity as a time point in which antibiotics usage was unavoidable for the health of themselves or their child. Respondents said that they would avoid using antibiotics unless necessary, and alternative methods were unavailable or ineffective for treatment. Mothers stated:

*“I would want to know that there was no other way and that antibiotics are necessary for whatever issue.”*

—PRG009

Children with severe infections or at risk of mortality were specific situations in which mothers understood that antibiotics were indispensable for the successful elimination of the disease. Half of the participants (*n* = 9) reported that they would be more concerned about the potentially fatal progression of a disease than the side effects of a medication. The following quotes portray women’s sentiments about how the severity of illness might affect their decision to use antibiotics:

*“If there was some kind of fatal infection that needed antibiotics. There are times when a medical intervention is necessary, and the antibiotics are helpful.”*

—PRG013

Interestingly, some mothers who initially reported their desire to avoid antibiotics or try alternative methods indicated that this sentiment only applied when mitigating mild infections. If symptoms worsened and their lives or the lives of their child were at risk, they would ultimately rely on antibiotics.

#### 2.2.6. Cues to Action

Cues to action were described as events that trigger women to take antibiotics for themselves or to give antibiotics to their children. The decision does not take into account perceptions of antibiotics and illness or women’s perceived expectations or threats to antibiotic use.

Participants (*n* = 7) described recommendations from physicians as a factor that would lead them to take antibiotics for themselves or give antibiotics to their child. Relying on the recommendation from their physician indicates a high level of trust in medical providers and supports the belief that physicians know best and would not do anything to harm women or their infants. The following statements broadly represent the sentiments that women expressed:

*“I usually trust doctors that they know what they’re doing. That they’ve gone to medical school and I haven’t—and if they would consider [antibiotics] to be necessary.”*

—PRG001

*“You know, like if your doctor says you need this, [you’re] gonna just do it. I wouldn’t ask any questions either, because it’s like your baby could die in there.”*

—PRG020

Notably, relying on the recommendation from their doctor to take antibiotics, women relinquished their role in the shared decision-making processes surrounding antibiotic usage.

### 2.3. Preferences for Antibiotic Counseling

Women were also asked about the extent to which they received antibiotics counseling during their pregnancy. Participant experiences with antibiotic counseling included any previous information they received from a healthcare provider about antibiotic use during and after pregnancy and for their infants.

Half of the participants (*n* = 9) reported that they had not received any information from their healthcare providers regarding antibiotic use during the pre- and perinatal period. However, when asked, all women expressed an interest in receiving information about the benefits and short- and long-term risks of antibiotic use for themselves and their children. Participants varied in their preference for the timing of antibiotics counseling; women were interested in receiving information about antibiotics upon finding out they were pregnant, during birthing classes, and after delivery. Most women were interested in receiving information about antibiotic use digitally, in the format of an email, and the information consolidated into a short summary or bullet pointed list. Participants overwhelmingly indicated that this information would influence their healthcare decision-making for themselves and their children. Statements coded regarding preferences for antibiotic counseling included the following:

*“I feel as soon as the mother knows she’s pregnant, they should start giving information about the different medicines.”*

—PRG006

*“Taking antibiotic information and emailing it out would definitely have a greater impact on my life.”*

—PRG014

*“Summary bullets points, these are the highlights.”*

—PRG018

*“Probably electronically, like email, and consolidate like a brief of a research report.”*

—PRG014

## 3. Discussion

To our knowledge, this is the first study to interview pregnant women about their perceptions, concerns, and conditions under which they would use antibiotics for themselves and their infants in the United States. We used the Health Belief Model as a framework to facilitate and characterize the understanding of mothers’ decision-making processes related to antibiotic use. Most women expressed understanding of the benefits of antibiotics; however, sentiments regarding antibiotic use were overwhelmingly negative. Women largely believed that antibiotics should only be used when necessary and not for preventative measures or in situations when other options are effective and available. Concerns that women had about antibiotic usage for themselves and their children included adverse effects to breastfeeding efficacy, microbial changes, and public health risks. Studies show that many women discontinue treatment after the confirmation of pregnancy due to perceived negative health and teratogenic risks of the medications [24,25]. Conditions under which antibiotics were more likely to be used included severe infection or threat of mortality, and necessity. Additionally, mothers stated that they would be more likely to give antibiotics to their child if they were born pre-term or required care in the NICU. Women largely emphasized that information about antibiotics contributes to their decision-making processes and asserted that more information should be given at large to pregnant women by healthcare providers during and after pregnancy, aligning with findings from previous studies [26].

This paper has several strengths and limitations. First, using the themes of the HBM, we were able to draw theoretical connections between mothers’ perceptions and opinions of antibiotics to characterize their decision-making processes. Thus, application of the HBM, the most widely used conceptual framework in health behavior research, strengthens our study by providing a framework to understand the complex factors that influence use and perceptions of antibiotics among mothers. One component of the HBM is to compare patients’ thought processes across several potentially modifying factors, including gender, age, parenting experience, ethnicity/race, and socioeconomics. Due to our small sample size, we were unable to detect meaningful differences between subgroups of our sample population. Additionally, we did not collect information about women’s race or socioeconomic status, which is a limitation. However, previous work suggests that perceptions of shared decision-making and self-efficacy surrounding health-related decisions during labor and delivery is variable among pregnant women of different race and socioeconomic backgrounds [27]. Our study population was comprised of women within typical childbearing years and of higher levels of education. As a primary goal of qualitative research is to identify patterns across the data, not to highlight differences, understanding perceptions and opinions of antibiotics among pregnant women, a high-risk, protected sample, is a strength. However, future studies with larger samples are warranted to identify potential differences in perceptions of antibiotics and preferences for antibiotic counseling among pregnant women with diverse backgrounds. Finally, we did not include time as a dimension when asking mothers about antibiotic usage. Timing in life and pregnancy may play an important role in healthcare decision-making, as family lifestyle can impact at what point women decide to seek treatment for themselves or their children. Subsequent studies should aim to understand how timing (i.e., first, second, third trimester, postnatal period medication effect on fetus) of decision-making for drug and antibiotic use for illness is considered among patients [28].

Our findings suggest important clinical implications for increasing awareness of pregnant women’s concerns of antibiotics and access to information on usage. First, many of the women reported receiving little to no guidance on proper antibiotic use during pregnancy or breastfeeding. All participants expressed an interest in receiving information about the benefits and short- and long-term risks of antibiotic use. The discrepancy between the lack of information provided, coupled with women’s desire to receive information about antibiotic usage underscores an important gap in health education and clinical practice. Since pregnant women are typically excluded from research aimed at examining the effectiveness of medications as well as premarketing medication studies, there is limited information on medication use and safety during pregnancy and teratogenic risk [29]. To fill this gap, there is a need for health education and interventions surrounding safe antibiotic use during pregnancy and postpartum that are tailored to meet the needs of women and the health literacy standards for equitable access across abilities [30]. Behavioral modification interventions are effective at reducing smoking among pregnant women [31] and increasing post-partum weight loss among breastfeeding mothers who are overweight and obese [32]. In particular, evidence from tailored health interventions effectively stop or reduce prenatal alcohol use among pregnant women [33] and increase pregnancy knowledge among women who are trying to get pregnant [34], making them appropriate strategies for educating women about antibiotics. Disseminating information based on the timing and through channels that women prefer is also an important consideration. Nonetheless, information on the effects of antibiotics on pregnancy outcomes continues to grow, and research findings can be used to inform interventions aimed at improving women’s knowledge on the effects of antibiotics during pregnancy [35].

Second, given the concerns and overall negative sentiments toward antibiotics use—women overwhelmingly expressed a strong desire to “avoid taking antibiotics”—there is a critical need to ensure information about antibiotics is not only accurate but perceived as credible among women who are pregnant and breastfeeding. Healthcare professionals (HCPs) (e.g., physicians) are important sources of health information among pregnant women, and women of childbearing age believe that HCPs should initiate conversations about the risks of medications [28]. The majority of obstetrician–gynecologists report that patients inquire about risks of both prescription and over-the-counter medications on their health and the health of the fetus, underscoring the important role of physicians as trusted sources of knowledge and information for women [36]. The overall positive sentiments toward physicians among pregnant women coupled with our findings that prescriptions from physicians would serve as cues to action for antibiotic use suggest the need for HCPs to address the risk-to-benefit ratio of antibiotic usage with pregnant and breastfeeding patients as part of clinical practice. Indeed, relying on HCPs to educate patients about appropriate antibiotic use may be challenging due to time and other constraints. Beginning the conversation about antibiotic use during initial appointments and providing access to additional electronic health education resources about antibiotics (e.g., via email or patient mobile applications or portals) would address participants’ desire to receive information about antibiotics in digital forms and provide a crucial link between patients and HCPs. In addition to providing information about antibiotics, increasing access to opportunities to ask questions may also contribute to women’s self-efficacy. SafeMotherMedicine, a Norwegian medicine information service for pregnant and breastfeeding women, is a website and telephone service that has answered over 30,000 questions between 2011 and 2020 mostly related to teratology information on medications [37]. Enhancing communication between patients and providers could also be achieved by the development of health education interventions that encourage women to proactively ask questions and discuss risks of medications with providers [26]. Shared decision-making tools and resources aimed to maximize patient–physician interactions by facilitating discussion of benefits and risks of antibiotic use during pregnancy are needed [38,39]. Finally, as many mothers showed a preference for alternative methods of treatment, HCPs and health education materials should address the limitations of using complementary or alternative medicine and indicate conditions in which medical intervention, such as using antibiotics, is necessary for treatment. Future research on strategies for developing and disseminating credible materials about appropriate antibiotic use are needed to educate women and health decision-makers and to ameliorate this potential public health concern. In addition to biomedical influences on decision-making, women are also influenced by their families, partners, and cultural and societal norms and expectations [15,40,41,42]. Despite receiving evidence-based information regarding the safety of medications, women also rely on internet resources [26,43]. Websites with information and guidance on medication safety during pregnancy supply inadequate and inconsistent information [44,45]. There is a need for future studies that seek to disentangle ecological factors that influence and drive medication decision-making.

## 4. Materials and Methods

### 4.1. Design and Aims

This qualitative study examines pregnant mothers’ perspectives and decision-making processes regarding antibiotic use for themselves and their children. We used semi-structured interviews to explore participants’ personal experiences, opinions, concerns, and conditions for antibiotic use during pregnancy and pediatric care. This is a sub-study of the Breastfeeding and Early Child Health (BEACH) clinical study conducted at the University of Florida.

### 4.2. Sample

The inclusion criteria were as follows: (1) women aged 18 to 45 years old (2) and who self-reported as currently pregnant. The exclusion criteria mirrored our parent study. The study excluded individuals with prior pregnancy complications or a previous history of substance abuse.

### 4.3. Recruitment

Recruitment for the study was dependent on the participants’ geographical proximity to the study site. Advertisements of the parent clinical study, in the form of fliers, were posted around Alachua County, Florida. Locations frequently visited by pregnant and breastfeeding women were targeted, such as hospitals, restaurants, and grocery stores. We screened all individuals who contacted us with interest in the study.

### 4.4. Data Collection

Between September 2017 and December 2018, experienced interviewers (MF, EFG) conducted eighteen private 30–60-min interviews. The interviewer used a semi-structured interview guide (see Table 2) to frame the interviews and ask about participants’ opinions and concerns about antibiotic use. The interviewer asked participants to share their experiences with antibiotic use and counseling during pregnancy. The guide also prompted discussion about women’s preferences for antibiotic counseling by healthcare providers. In addition to the interview data, we used a structured survey to gather sociodemographic information for each of the participants that included age, parity, education, and employment status. The interviewer audio-recorded all interviews with the participant’s consent, and the audio recordings were professionally transcribed (Datagain, Secaucus, NJ). The University of Florida Institutional Review Board approved the protocol for the study (NCT03036696).

### 4.5. Data Analysis

We entered the audio transcripts into NVivo 12 Plus [46], which is a qualitative data management program. One trained coder (L.Y.C.) conducted the initial coding and categorization of the data. Analysis began with a thorough reading of the interview transcripts to gain familiarity with the data. Research team members read all the transcripts line by line and identified and compared important content. We grouped similar content that emerged and coded the text into a descriptive theme, which we termed inferential themes.

We organized the HBM themes according to women’s perceived expectations and threats [47] (see Table 3). Women’s expectations regarding antibiotic use included perceived benefits of antibiotic treatment, perceived barriers to using antibiotics, and perceived self-efficacy about antibiotic use, including the ability to exercise control over antibiotic use. Women also identified perceived susceptibility to illness and perceived severity of illness as threats to taking antibiotics. We identified “cues to action” as specific external events (such as an antibiotic prescription from a health care provider) that trigger the action of taking antibiotics and are not associated with perceived benefits or barriers to taking antibiotics, or perceptions about the severity of illness [47]. L.Y.C. categorized the inferential themes into the framework of the Health Belief Model. We included all themes in interpretation and results, regardless of whether they fit in the framework. After this, a second coder (A.H.) independently coded the transcripts based on the HBM framework codes. Then, L.Y.C. and A.H. met and discussed any discrepancies in coding until they reached consensus.

## 5. Conclusions

We used the HBM and qualitative methods to explore pregnant women’s perceptions, concerns, and conditions under which they would use antibiotics for themselves and their infants. Women described the benefits and barriers to taking antibiotics and cited antibiotic prescriptions from healthcare providers as a cue to action that would inform their health decisions. In addition, all participants expressed an interest in receiving information about the benefits and short- and long-term risks of antibiotic use. Given that 1 in 4 healthy, full-term infants are prescribed antibiotics during their first three years of life, regardless of delivery type [48], future research and health education interventions are needed to optimize antibiotic use, knowledge, and decision-making.

## Figures and Tables

**Table 1:** Participant demographic information.

Characteristic	Frequency (*n*)	Percent (%)
**Maternal age**		
20–30 years	8	44
31–40 years	10	56
**Previous children**		
Yes	7	39
No	11	61
**Highest level of education**		
Professional/Graduate degree	9	50
College degree	5	28
Associate/Technical/Vocational degree	3	17
High school degree	1	5
**Antibiotics during pregnancy**		
Did not receive	12	67
Received during current and/or previous pregnancy	5	28
Missing	1	6
**Antibiotic counseling during pregnancy**		
Received some counseling	2	11
Received counseling	5	28
Did not receive counseling	9	50
Missing	2	11

**Table 2:** Interview guide.

**Previous experience**	- Have you taken any antibiotics during this pregnancy? Any reasons why or why not? - If you had a situation where you need antibiotics, would you take it? - Did your doctor give you information about antibiotics while pregnant? - Has your doctor given you information about providing antibiotics after delivery?
**Opinions of antibiotics**	- Do you think antibiotics during pregnancy is beneficial or non-beneficial? - Do you think taking antibiotics after delivery will be beneficial or non-beneficial to your baby? - Do you think routinely giving antibiotics to infants during the first year of life is beneficial or not beneficial?
**Concerns/risks**	- Do you think antibiotics during pregnancy will impact breastfeeding goals? - Do you have concerns about your baby getting antibiotics after delivery? - Are there any concerns with antibiotics that you think researchers or clinicians should focus on and why?
**Antibiotics counseling**	- As a parent, would you be interested in information about short-term and long-term effects related to antibiotics? - What type of information do you think you would be interested in receiving about antibiotics? - In what format would you want to receive this information? - How do you think this information would impact the healthcare choices about your infant? - Should the health system prioritize getting information about antibiotics utilization to parents? - When is the best time to receive information on antibiotics utilization?

**Table 3:** The Health Belief Model: theme organization and descriptions.

Category	Construct	Description
Perceived Expectations	Perceived benefits	The belief that antibiotics will successfully reduce the risk and severity of infection/illness.
	Perceived barriers	Perceived negative effects and costs of antibiotics.
	Perceived self-efficacy	The conviction that one can take action and/or make a decision about taking antibiotics.
Perceived Threats	Perceived susceptibility	Beliefs about the susceptibility of the mother or infant to an infection or disease that requires antibiotics.
	Perceived severity	Perceived severity of a condition and its clinical sequela.
Cues to Action		Events that trigger one to take antibiotics.
